# Imaging in the Late Time Window of Acute Ischemic Stroke: Enough Is Enough?

**DOI:** 10.1007/s00062-025-01504-9

**Published:** 2025-02-17

**Authors:** Jessica Jesser

**Affiliations:** Dept. of Neuroradiology, University of Heidelberg, INF 400, 69120 Heidelberg, Germany

Imaging acquisition and interpretation for acute stroke should be as straightforward as possible to ensure widely accessible use and quick decision-making for patients worldwide. This applies to both primary care centers and comprehensive centers as well as to higher- and lower-income countries. In the later time window of stroke, i.e. > 6 h after symptom onset, most current guidelines, such as those from the American Heart Association/American Stroke Association (AHA/ASA) in 2019, the European Stroke Organization in 2023 and the Chinese Stroke Association in 2023 [1–3], propose an advanced imaging protocol for acute ischemic stroke patients. However, in primary care centers or lower-income countries perfusion imaging—primarily recommended for advanced imaging—may not be feasible due to the increased costs or a lack of expertise in conducting and interpreting perfusion imaging. Perfusion imaging is not generally standardized and data analyzing methods are variable. Interpreting perfusion results can lead to discussions between radiologists and neurologists and cause unnecessary delays in decision-making.

In our current edition, a study by Shchehlov et al. shows that imaging criteria based solely on non-contrast CT (to assess infarct extent) and single-phase CT angiography (to assess for large vessel occlusion and collaterals) yield outcomes comparable to those obtained using the more complex perfusion-based criteria defined by the study inclusion criteria of the DAWN and DEFUSE-3 trials in 2018 [4, 5]. According to the results of Shchehlov et al., the following criteria for indication of endovascular therapy can be applied without using CT perfusion for patients between 6–24 h after stroke onset: large vessel occlusion, ASPECTS > 4, good collateral status. The study results indicate that non-contrast CT and CT angiography without perfusion might be a sufficient and also more standardized and easily accessible imaging modality for stroke patients in the later time window.

Upcoming sub-analyses of last year’s large core trials, including the TENSION trial [6] will show whether this also applies to larger core strokes.
